# Using the water footprint concept for water use efficiency labelling of consumer products: the Greek experience

**DOI:** 10.1007/s11356-022-23573-w

**Published:** 2022-10-15

**Authors:** Ioanna Nydrioti, Helen Grigoropoulou

**Affiliations:** School of Chemical Engineering, National Technical University of Athens, 157 80, Athens, Greece

**Keywords:** Water footprint, Water labelling, Consumer preferences, Water use efficiency, Greek survey, Green marketing driver

## Abstract

Freshwater is crucial for food supply, as irrigation water and as production or incorporated water in industrial production of consumer goods (e.g. food, cosmetics). Manufacturing industries follow different protocols and receive different certifications for water management and efficiency in their plants, which are also labelled on the packaging. Nowadays, consumers appear to be increasingly concerned about environmental challenges, therefore many sustainability labels have been developed (carbon, water, ecological footprint) to facilitate consumers to make more sustainable choices concerning their purchases. Consumers’ behaviour towards carbon footprint labels has been thoroughly examined in international literature; however, WF labelling studies are very limited. The key to water efficiency labelling in consumer products could be the water footprint (WF), as it measures the total volume of freshwater used to produce a product, over the full supply chain, including virtual water, “hidden” in the products, services and processes. The implementation of WF labelling of consumer products in Greece was investigated, using a questionnaire with demographical data and statements on water sustainability. The results indicate that younger consumers are not quite aware of environmental challenges related to water. According to the findings, WF could be an effective marketing driver towards water sustainability since consumers prefer a quantified label concerning water consumption, regardless of their educational level, and they are willing to pay an extra price for water sustainable products even if they have an unstable job. Manufacturers could then promote their sustainable profile and performance effectively by adopting a WF relevant labelling.

## Introduction

Water is an essential resource to sustain life and development, being a key element of both natural ecosystems and production activities. Freshwater is crucial for food supply, as irrigation water and as production or incorporated water in industrial production of consumer goods (Sala et al. 2013). However, despite its readily acknowledged importance, water resources management is often insufficient leading to numerous environmental challenges. The main drivers for water overexploitation and pollution are population growth and economic development through consumption of water-intensive goods and services (Hogeboom 2020). Population increase leads to augmented consumption of goods and services requiring water for their production. Climate change is also affecting water use; extreme weather events and warmer temperatures raise water demand in agriculture, industries and households.

Industries are expected to comply with the principles of sustainable development, focusing on broad water accounting in production and distribution so as to better manage and reduce their freshwater consumption. Currently, manufacturing industries in Greece follow different protocols and receive various certifications for water management and efficiency, usually labelled on the products and/or used in marketing campaigns to highlight the environmental consciousness of the company.

The recent years, consumers worldwide have shown to be increasingly concerned about the environment and the impacts of their daily life to it and become, over the years, more aware of environmental challenges and needs (Liu et al. 2017). Therefore, in the last 10 years, many sustainability labels have been developed to facilitate consumers to make more sustainable choices concerning their purchases (Grunert et al. 2014). The goal of sustainability labels is to provide companies with a marketing tool so as to communicate their sustainability practices concerning energy consumption, water consumption, ethical trading, etc. to consumers (Kimura et al 2010). To this end, footprint labelling (carbon, water, ecological) is also part of these efforts with the aim of providing consumers understandable information on the environmental impact of the product they choose (Rondoni and Grasso 2021). Consumers react positively to the inclusion of footprint labels on consumer products, as 72% of European citizens support their introduction and agree that it should be mandatory; however, there is poor understanding on consumers’ reactions and preferences concerning sustainable information presented by footprint labels as well as the factors that influence their perceptions and choices (Liu et al. 2017).

Many other sustainability labels have been introduced in consumer products such as the green dot trademark, the fair trade certification label and the organic label concerning other aspects irrelevant to water consumption. Extensive research has been conducted on carbon footprint (CF) labelling over the last decade with numerous contributions in scientific journals concerning consumer preferences and perceptions on CF labels, their willingness to pay for CF labelling and the drivers for choosing products with these labels (Rondoni and Grasso 2021). Finally, ecological footprint (EF) studies have been conducted to examine consumer choices and their willingness to pay mainly for agricultural (Mamouni Limnios et al. 2016; Patrizi et al. 2018) and seafood products (Altiok et al. 2021). Although the consumers’ drivers for footprint labelling and their willingness to pay could potentially show similarities among various footprint labels, very limited studies and consumer preferences surveys have been performed for water footprint (WF) labels (Grebitus et al. 2016).

The aim of this research paper is to present the state of the art of CF labelling research as well as the results of published studies on EF labelling and the current applications on water labelling in consumer goods. Then, the factors that influence the adoption of a WF label in Greece are examined based on consumers’ environmental awareness, knowledge and preferences on water policies and labelling and willingness to pay for sustainable products combined with certain demographical characteristics. Finally, common results and conflicts are identified with the studies for CF labelling, so as to enable the development of a unified strategy for footprint labelling on products.

## Consumers’ pro environmental behaviour towards CF and EF labels

Nowadays, one of the most recognizable sustainability label is the CF label. The CF labelling concept was initially developed in 2007 in the UK and defined as “a measure of the total emission of carbon dioxide (and other greenhouse gases such as nitrous oxide and methane) caused by a particular product throughout its life cycle” (Thøgersen and Nielsen 2016). Since 2007, CF labelling is rapidly implemented on the products, so many studies and consumer preferences surveys have been conducted in different countries such as Finland, Sweden, China, UK and Italy to explore consumers’ perceptions on CF labels, attitudes towards sustainability purchases, whether or not consumers seek information about products’ sustainable manufacturing on the packaging and if they are willing to pay more for sustainable products (Rondoni and Grasso 2021; Wong et al. 2020; Hartikainen et al. 2014; Moser 2016; Steiner et al. 2017; Laureti and Benedetti 2018; Grebitus et al. 2015; Gadema and Oglethorpe 2011; Zhao et al. 2018; Van Loo et al. 2014; Canavari and Coderoni 2019).

As presented in the review paper of Rondoni and Grasso (2021), several studies on consumers’ behaviour towards CF-labelled food examined the demographical characteristics of consumers (e.g. age, gender, education), their attitude and concern on environmental challenges, their tendency to read CF labels on products, their understanding of CF labels, their willingness to pay, their food purchasing habits and the alternative design and positioning of the label on the packaging.

Age seems to influence the acceptance of CF labels as older people are more aware of environmental challenges which increases their environmental concern (Wong et al. 2020; Hartikainen et al. 2014). The awareness of consumers in many studies (Moser 2016; Steiner et al. 2017; Laureti and Benedetti 2018) was examined through a self-reporting exercise in which consumers reported their level of concern for environmental conditions. The findings indicate that high scores in self-reported awareness support that the higher the environmental sensibility, the more positive acceptance of CF-labelled goods (Grebitus et al. 2015).

Concerning information seeking, consumers are not willing to search and read CF labels mainly because they do not quite understand them. Academic studies show that the drive of consumers to check for CF labels and their knowledge and understanding of the labels could influence radically their decision making (Rondoni and Grasso 2021; Gadema and Oglethorpe 2011). Nevertheless, most consumers self-reported difficulties on understanding and interpreting CF labels, which limit the purchase of CF labelled products (Kimura et al. 2010; Gadema and Oglethorpe 2011).

Income affects critically the decision of consumers to purchase CF-labelled products, as people with high income are more willing to pay a higher price for products with CF labels (Zhao et al. 2018; Van Loo et al. 2014; Canavari and Coderoni 2019). Also, the type of the product affects the willingness to pay, as the more necessary the product for the household (e.g. milk, beef), the higher the willingness to pay for that product (Rondoni and Grasso 2021).

Existing academic literature concluded that the adoption of a commonly accepted CF label in each industrial branch my manufacturing companies could increase the consumers’ understanding of CF labels and therefore influence their purchasing habits concerning sustainability (Van Loo et al 2014; Rondoni and Grasso 2021). Also, consumers’ acceptance of low CF products could be enhanced by large promotional campaigns and educational programs (Rondoni and Grasso 2021).

Ecological footprint (EF) is a sustainability indicator of resources’ exploitation and pressure by human activities (Yu et al. 2019). The indicator measures the populations’ use of natural resources, i.e. cropland, marine ecosystems, grazing land, forest products, build environment and carbon (Borucke et al. 2013; Lin et al. 2015; Wackernagel and Rees 1996), so EF labelling enables consumers to examine the ecological impact of products in terms of materials use and energy and transport in the production and distribution phases. Despite that EF is a holistic ecolabel, very limited studies have been conducted to examine consumers’ behaviour towards this label as its use is more restrained, compared to CF labels.

In EF studies for fruit purchases, the willingness to pay of consumers is elevated for EF-labelled products. The sociodemographic factors that influence their choices are (a) mainly the gender, as females are more aware of environmental issues and tend to have a healthy diet while (b) education and (c) income do not affect their choices (Mamouni Limnios et al. 2016). Concerning Mediterranean seafood products, consumers in Italy, Turkey and Croatia are willing to seek information on the product label and acquire more information concerning products’ sustainability from the seller (Altiok et al. 2021).

CF and EF labels have been placed on products to demonstrate their sustainability concerning CO_2_ emissions and pressure on natural resources generated by products’ manufacturing, respectively. Many studies on the pro environmental behaviour of consumers towards CF and EF labels have been conducted so as to understand consumers’ choices based on sustainability labels. On the other hand, the WF label has not received as much attention compared to CF and EF labels. The introduction of water use efficiency labels, such as the WF label, could increase consumers’ engagement to sustainability products, as water is essential in their everyday life.

## Water use efficiency labelling

### Water stewardships and certifications

Large manufacturing industries in Greece apply sustainable water management practices in their plants so as to acquire different stewardships and certifications, such as European Water Stewardship, Alliance for Water Stewardship and TRUE Certification. European Water Stewardship, actually adopted by Coca Cola HBC and BIC (European Water Stewardship 2012), sets 4 principles, 15 criteria and 49 indicators aiming to map, grade and evaluate the water management practices within the boundaries of an industrial plant. The Alliance for Water Stewardship, actually adopted by Coca Cola HBC and Nestle (Alliance for Water Stewardship 2019), proposes a 5-step framework for sustainable water management in the production site and its local water catchment, including indirect water use in the supply chain. Each step consists of plenty of criteria to be met, each one comprised of one or more indicators: “core” indicators, representing minimum requirement, and “advanced” indicators, to achieve higher levels of water stewardship status and to promote continual improvement. The inclusion of a production plant to the Alliance Water Stewardship (Alliance for Water Stewardship 2019) has also been used in Greek promotional campaigns (Nestle Waters Hellas 2017) to enhance the environmental image of a company. Finally, TRUE certification, recently acquired by Colgate-Palmolive, (Green Business Certification 2020), ensures that the product was manufactured in a zero-waste plant.

While sustainable water management in an industry is vital, it is also crucial to encourage changes in consumer’s behaviour regarding sustainable production and consumption of water-intensive goods (Grebitus et al. 2016). Certifications and stewardships are also labelled οn products and/or used in promotional campaigns (Nestle Waters Hellas 2017), to disseminate the environmental awareness of the company. Nevertheless, these labels cannot be considered an adequate green marketing driver since they do not enhance consumers’ understanding of “how much they contribute to the water consumption, water pollution and water scarcity in different places”, neither support them to compare similar products and make conscious choices based on water consumption and the impacts on water resources throughout the hole production cycle from supply chain to the end-user (Manson and Epps 2014).

### Water footprint as an environmental marketing driver

The key to water efficiency labelling in consumer products could be the water footprint (WF), which is a multidimensional indicator of volumetric water use and pollution. It measures the amount of water used to produce each good and service we use (Hoekstra et al. 2011). Indirect water use is also included in the indicator (e.g. water needed for energy production, which is used in a specific manufacturing plant) (Manson and Epps 2014). It has to be noted that most certifications and stewardships do not include in their accounting supply chain water and virtual water. WF could then be an effective marketing driver as it could give the consumer a quantified measurement of water consumption during processing and supply chain, leading him to choose products with lower water usage.

Several studies worldwide have examined consumers’ choices, showcasing that consumers are willing to pay an extra price for sustainable products as they become more aware of sustainable production and choose mostly environmentally friendly products over others (Grebitus et al. 2016; Simeonidou and Vagiona 2018). A “WF label” could be an effective solution for promoting sustainability. It is based on the footprint concept (e.g. carbon footprint label), which have gained wide public acceptance, and could enable companies in advertising their sustainable practices effectively and consumers in choosing between products according to their water sustainable production.

In many countries, especially in Scandinavia, companies launch products with a WF label, showing water consumption per product weight (e.g. 100 L/100 g product) and the relative distribution of water consumed among supply chain, manufacturing and packaging (Manson and Epps 2014). Although a specific benchmark WF for each product type is not presented on the packaging so as to offer a basis for conscious choice, a common policy on water labelling could enable consumers to choose among similar products. To this end, the International Organization for Standardization (ISO) published an international standard describing principles, requirements and guidelines for the quantification and reporting of WF, i.e. ISO 14046 (ISO 14046,2014). Very few companies have already adopted ISO 14046, probably because on the one hand, ISO standards are voluntary and on the other hand, each process and supply chain has its own characteristics and needs a customized approach on WF quantification. As a result, its implementation scale is so far very limited (Forin et al. 2020). Therefore, the espousal of a common WF label on the products for each industrial branch could be enhanced by the development of a WF estimation framework customized for the needs and the characteristics of each branch as well as by the enactment of international policies for labelling water consumption and pollution on the packaging of consumer products.

In Greece, for the time being, product labelling concerning sustainable water management is very limited and focuses on presenting the stewardships and certifications in a packaging label. To our knowledge, there are not any studies which involve consumers performed in Greece, examining their choices of particular products based on sustainability in different educational, financial or sustainability awareness contexts. The present study tends to analyse the influence of socio-demographics factors on water-related environmental awareness and on consumers’ choices towards environmentally friendly products as well as to examine consumer preferences based on the sustainable use of water in the production of consumer goods.

## Materials and methods

**Fig. 1:**
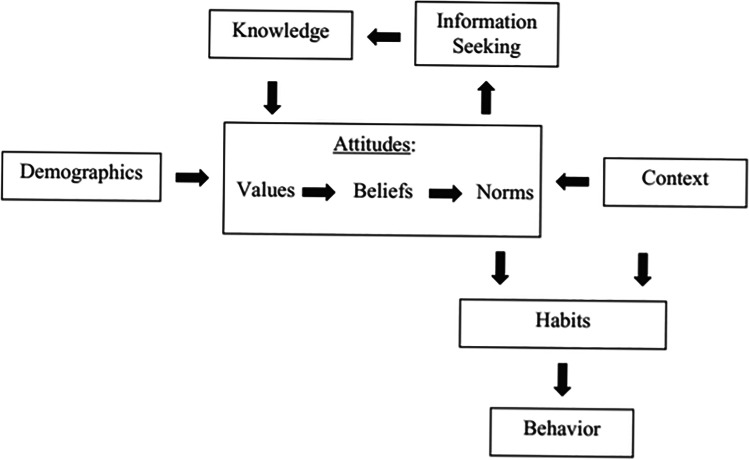
Conceptual framework of alphabet theory (Zepeda and Deal 2009)

To examine the pro environmental behaviour of consumers towards WF labelling, a public opinion survey was developed based on the alphabet theoretical framework proposed by Zepeda and Deal (2009) (Fig. 1). The alphabet theory has been already used by many researchers for studies that examine consumers’ behaviour concerning products’ sustainability (Feldmann and Hamm 2015; Schaufele and Hamm 2017; Rivaroli et al. 2020; Stampa et al. 2020), as it includes socio-demographic characteristics, attitude and habits, knowledge, information seeking and purchase context (Vecchio and Annunziata, 2012; Van Loo et al. 2017).

A public opinion survey was carried out through a questionnaire, distributed to consumers and uploaded in social media platforms from March to May 2021. The responders were 326 consumers living in Greece. The questionnaire was divided in two sections. The 1st section collected demographical data and data on education level of consumers, presented in Table 1, as consumers’ age, gender, education and job stability affects their behaviour (Canavari and Coderoni 2020; Grunert et al. 2014; Hartikainen et al. 2014; Koistinen et al. 2013). The set of demographical data required was based on relevant guidelines for sociodemographic research (Hoffmeyer-Zlotnik and Warner 2018).

**Table 1:** Demographical data required from the participants

Gender	Age	Total number of people living in the house	Educational level	Work status	Residence
• Female • Male	• 18–29 • 30–39 • 40–49 • 50–59 • 60–67 • 68 +	• 1–2 • 3–4 • 5 +	• High school graduate • Graduate of Institute of Vocational Training (IVT) • University • Master/Ph.D	• Unemployed • Student • Farmer • Full time job • Part time job • Permanent Employee • Entrepreneur • Retired	• Athens • Thessaloniki • Other

The 2nd section was developed on Likert scale, a psychometric scale proposed by the American social scientist Rensis Likert in 1932, used in questionnaires to assess the degree of agreement/disagreement of participants regarding various statements. Usually, there are 5 answers on an ordered scale and participants are asked to choose the one that best suits them (Kyriazi 1998). In the current study, statements concerning water sustainability, water efficiency labelling of consumer products, the WF, the need for quantified labels concerning water consumption and the willingness to pay more for water sustainable products were included (Table 2) and the potential answers were “Definitely yes”, “Probably yes”, “I do not know”, “Probably no” and “Definitely no”.

**Table 2:** Statements of the survey

Q1	I consider myself aware of water use
Q2	In my daily life I try to save water
Q3	I recognize that water will gradually become a non-renewable natural resource
Q4	I recognize the importance of saving water in all sectors of the economy (e.g., industry)
Q5	I know that the packaging of many consumer products states the company’s water policy
Q6	I would search information about the company’s water policy that I read on the packaging
Q7	I would prefer to see on the label the water needed for the production of the product (e.g., 20 L/product) instead of the company water policy
Q8	I know what is the water footprint of a process or a product
Q9	I am willing to pay 5% more for my shopping cart so that the products I choose are produced with low water use
Q10	I take into account the environmental image of the company in the product that I buy
Q11	I would choose an environmentally sensitive company for my purchases even if it has more expensive products

Following the alphabet theory, the statements were divided in three main categories:

i) *The level of environmental awareness of consumers*, as the higher consumers’ awareness towards environmental needs, the more willingness to be informed by WF labels (Canavari and Coderoni 2019; Grebitus et al. 2015; Van Loo et al. 2015).
ii) *Their knowledge and preferences on water policies and labelling*, as consumers with knowledge about WF indicator influences positively their behaviour (Canavari and Coderoni 2019; Feucht and Zander 2018; Guenther et al. 2012; Onozaka and McFadden 2011; Zhou et al. 2019) and different labelling of water use potentially influences their engagement.
iii) *Their willingness to pay for sustainable products*, as consumers who usually take into account the environmental practices of the production company in their purchases are more inclined to pay more for WF-labelled products (Canavari and Coderoni 2019; Gadema and Oglethorpe 2011; Roos and Tjarnemo 2011; Vecchio and Annunziata 2015).

The allocation of the statements of the survey into the three categories is presented in Table 3. A validity assessment has been conducted for the classification of the statements into the categories, achieving both construct and content validity as the statements and their categories have been used in similar pro environmental behaviour studies on sustainability labels (Canavari and Coderoni 2019; Grebitus et al. 2015; Van Loo et al. 2015; Feucht and Zander 2018; Guenther et al. 2012; Onozaka and McFadden 2011; Zhou et al. 2019; Gadema and Oglethorpe 2011; Roos and Tjarnemo 2011; Vecchio and Annunziata 2015) and they are highly representative of the qualities they are trying to measure.

**Table 3:** Classification of the statements of the survey into three categories

Q1	**Level of environmental awareness**
Q2	
Q3	
Q4	
Q5	**Knowledge and preferences on water policies and labelling**
Q6	
Q7	
Q8	
Q9	**Willingness to pay for sustainable products**
Q10	
Q11	

A cross-sectional data analysis was conducted to explore possible interlinks between the selected demographical groups and the responses to the survey statements. Based on relevant consumer behaviour studies on CF labels (Rondoni and Grasso 2021), it was expected that (i) age could influence the level of environmental awareness, (ii) knowledge on water policies and labelling is dependent to educational level and (iii) work status affects the willingness to pay for sustainable products.

## Results and discussion

### Consumers demographical characteristics

**Fig. 2:**
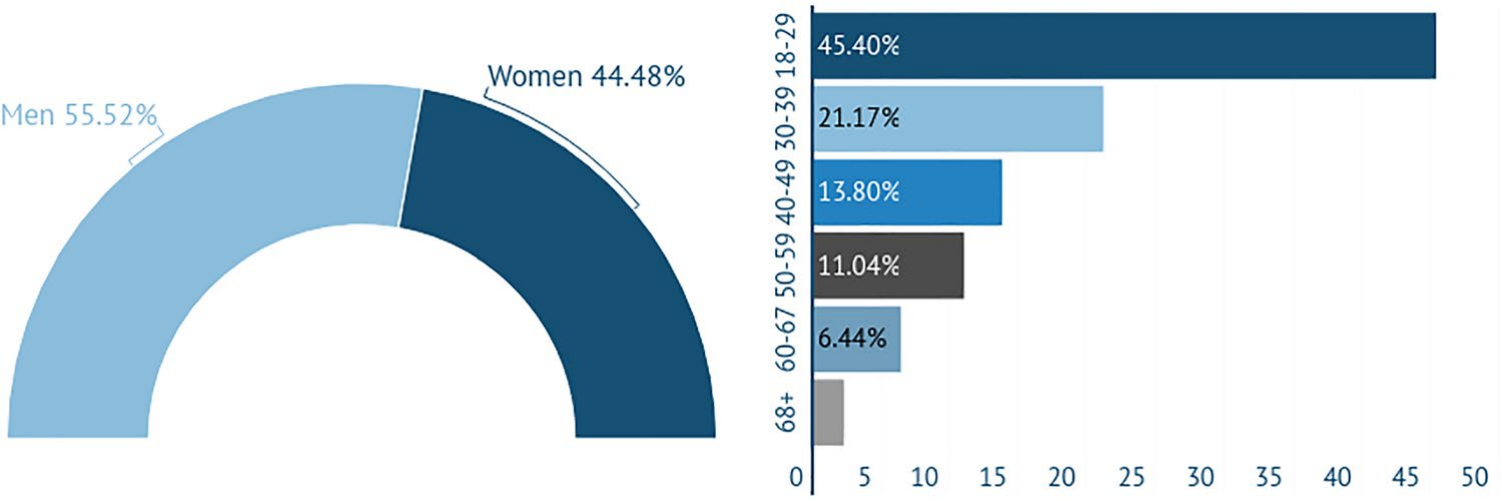
Gender and age characteristics of the participants

The participants of the survey were 181 women (55.52%) and 154 men (44.48%). The majority of them were between 18 and 39 years old (Fig 2).

**Fig. 3:**
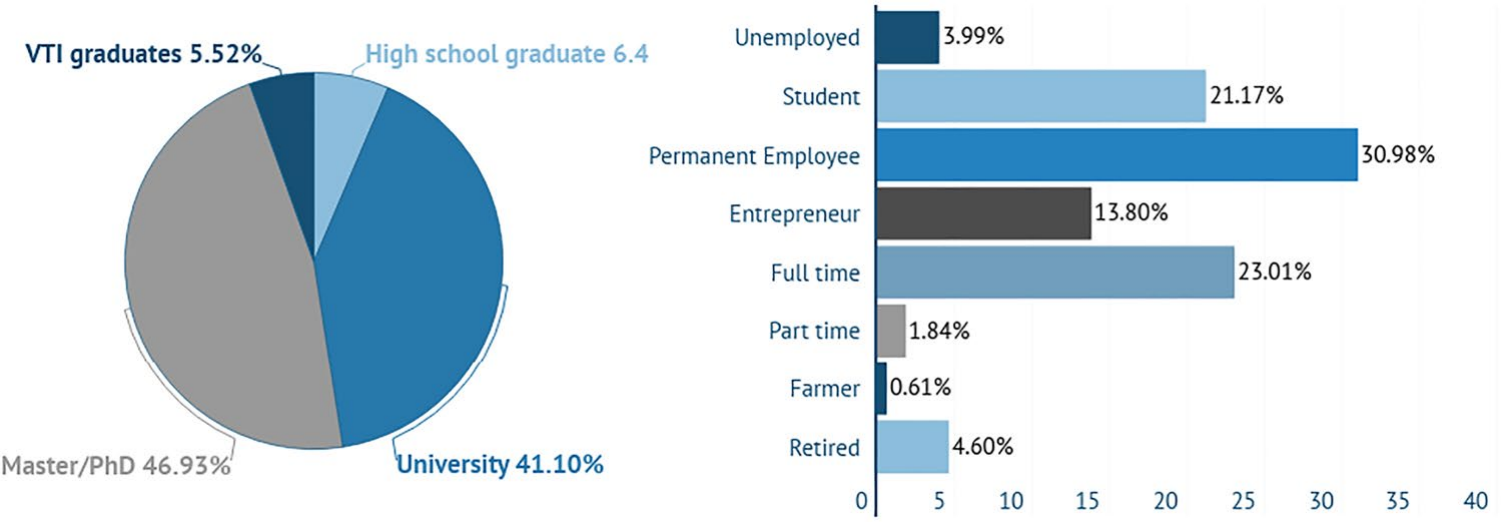
Educational level and work status of the participants

Ιn Fig. 3, the educational level and work status of the participants is presented. Most of them are highly educated, with Master Degree/Ph.D (46.93%) or having graduated from University (41.10%). Their education level is also affecting their work status, as the greater part of them are permanent employees (PE) (30.98%), entrepreneurs (13.80%) or have a full-time job (23.01%).

**Fig. 4:**
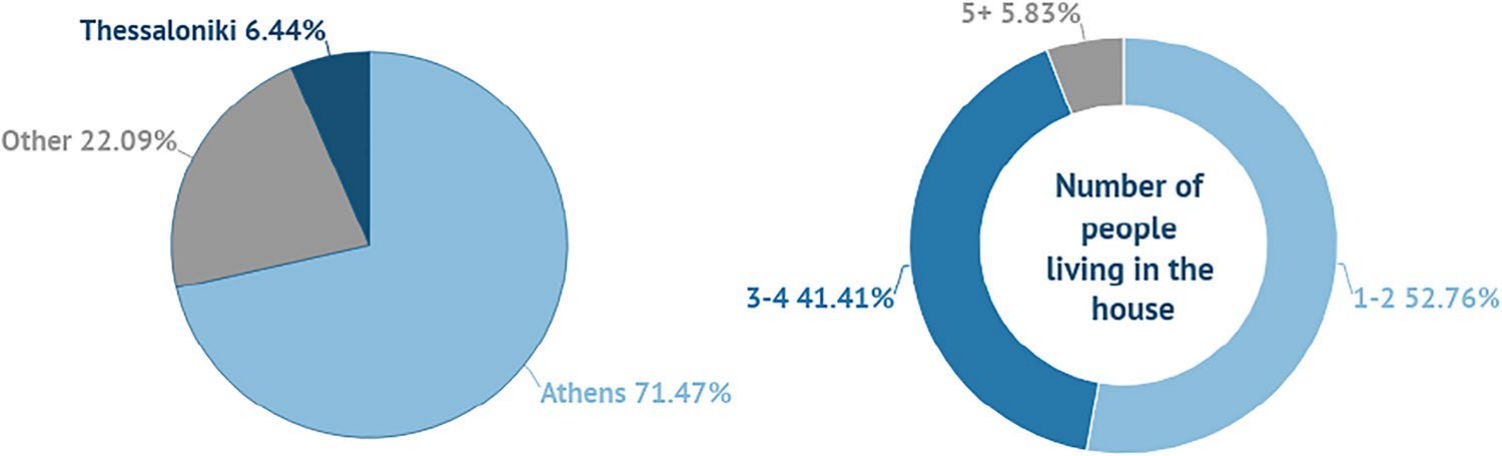
Residence of consumers and number of people living the house

In Fig. 4, the residence and the number of people living in the house is presented. The majority of the consumers live in Athens (71.47%), alone or with an additional person (52.76%).

### Consumers’ responses on the survey statements

Examining the level of environmental awareness of consumers, their knowledge and preference on water policies and water labelling and their willingness to pay for sustainable products the results of the survey, are analysed on the basis of the total participants.

**Fig. 5:**
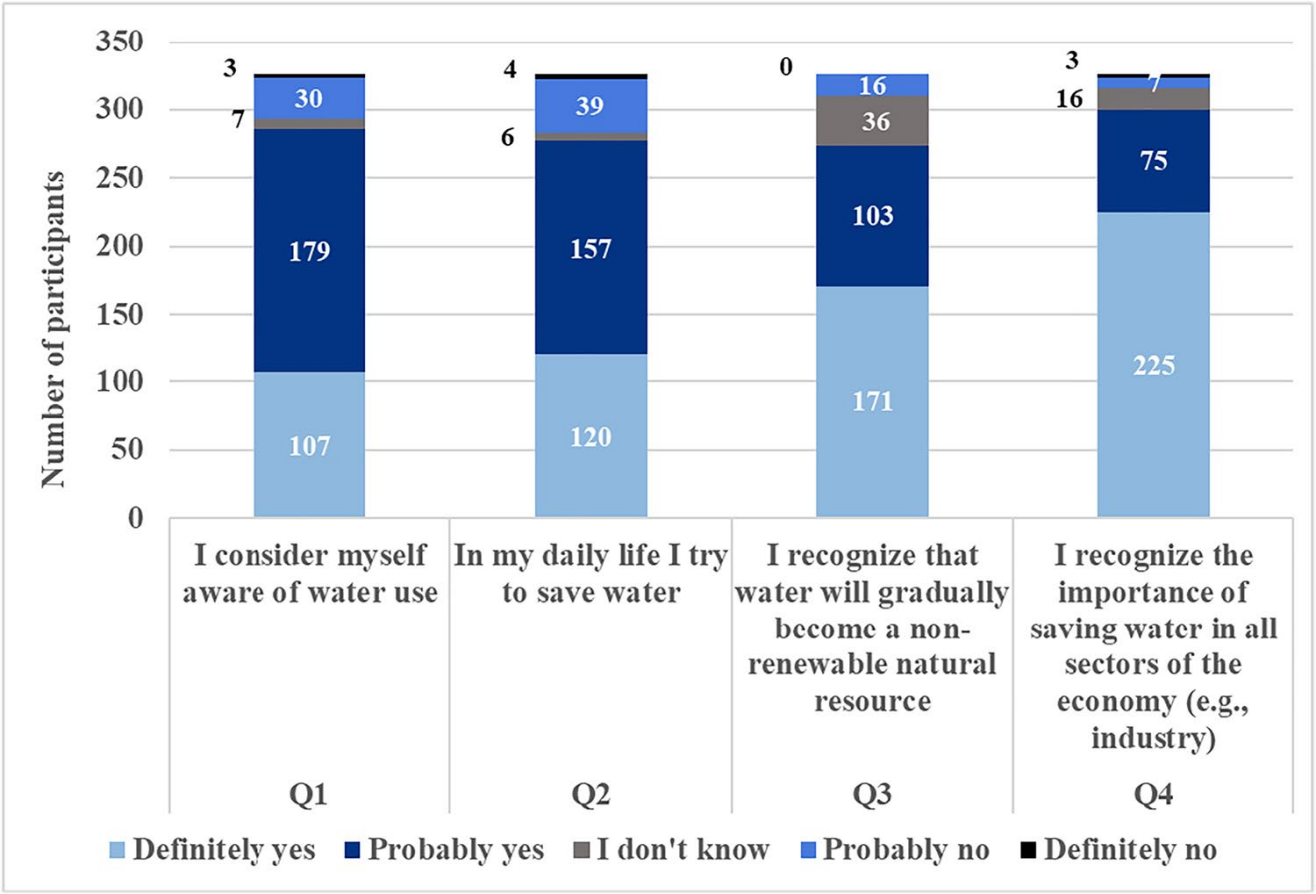
Consumers’ responses on statements Q1, Q2, Q3 and Q4

In Fig. 5, the detailed results for Q1, Q2, Q3 and Q4 statements are presented. The majority of the participants stated that they are aware of water savings and recognize the importance of sustainable water use in industry. For all 4 statements concerning environmental awareness, the responses of “Definitely yes” and “Probably yes” were above 84%. Almost 85% of the participants try to save water in their everyday life and 92% recognize the importance of saving water in all sectors of the economy.

**Fig. 6:**
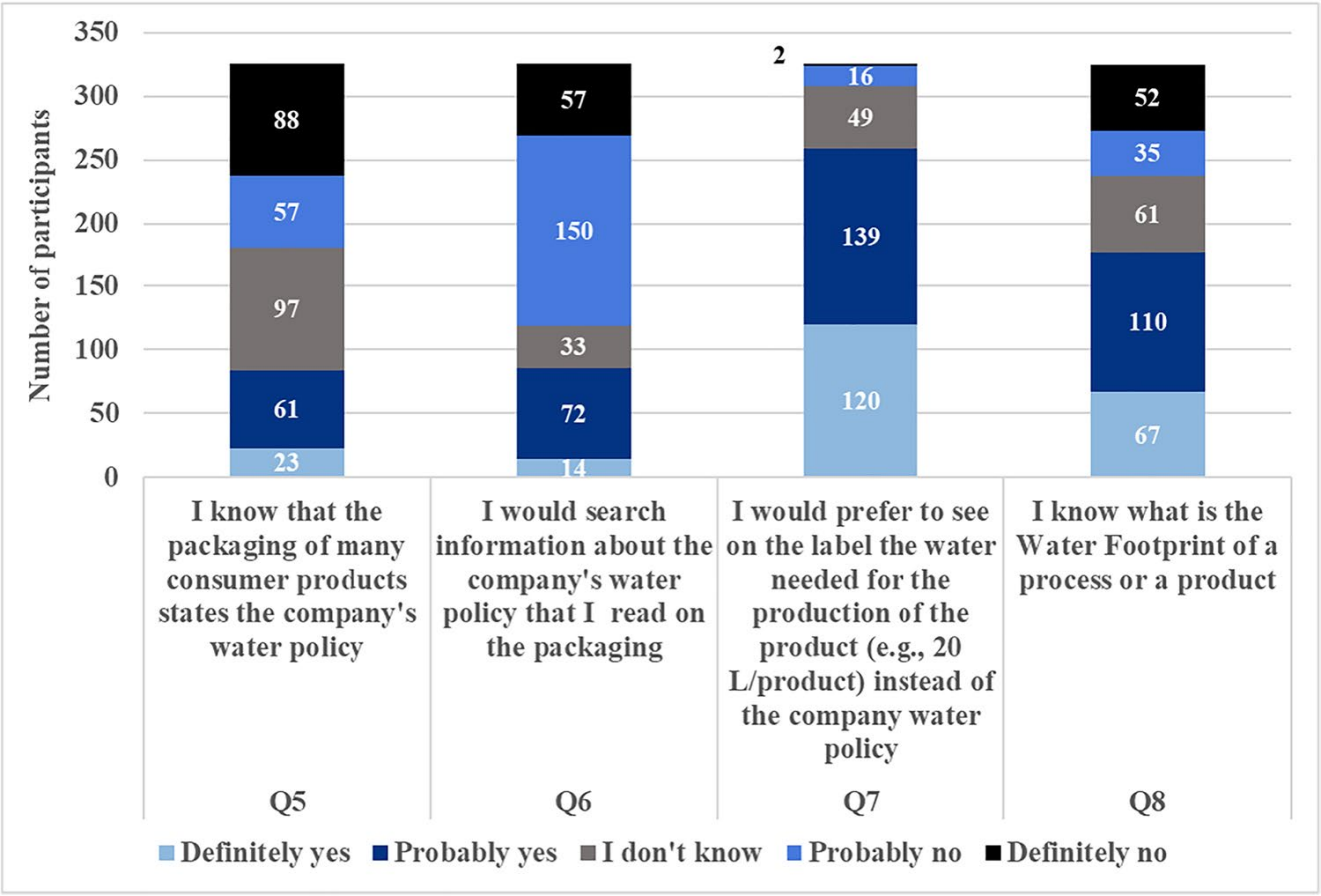
Consumers’ responses on statements Q5, Q6, Q7 and Q8

In Fig. 6, the consumers’ responses on statements Q5, Q6, Q7 and Q8 are presented. Although most participants responded that they try to save water in their everyday life, only 26% know that the packaging of many consumer products states the company’s water policy and would search additional information about it. Most of them (79%) would prefer to see on the label the water needed for the production of the product (e.g. 20 L/product) instead of the company water policy, while almost half of the participants (45%) do not know what is the WF of a product or a process. Therefore, the diffusion of the WF as environmental indicator to the public is critical for the comprehension of the concept and companies could effectively include it in products labelling.

**Fig. 7:**
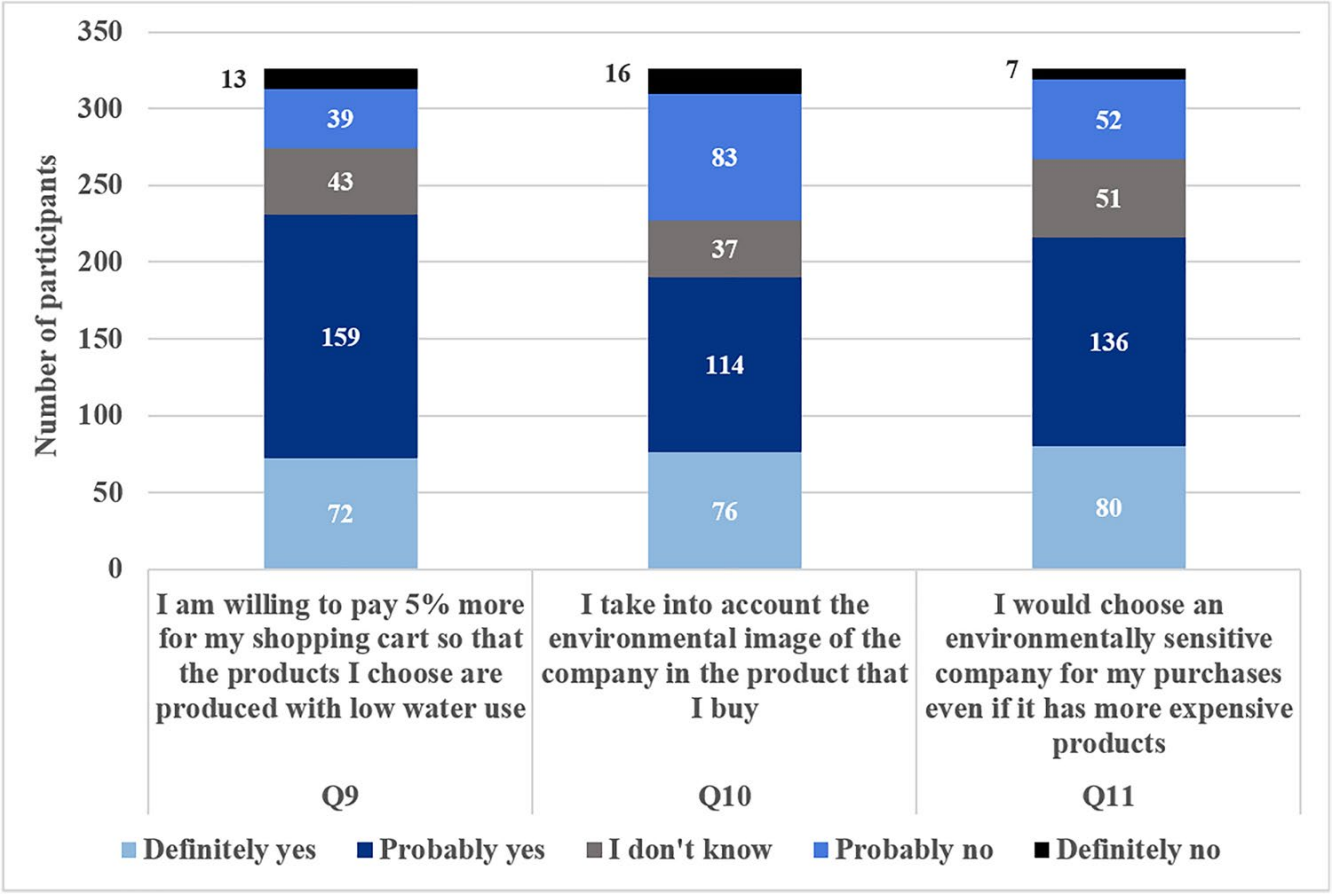
Consumers’ responses on statements Q9, Q10 and Q11

In Fig. 7, the consumers’ responses on statements Q9, Q10 and Q11 are presented. The current study confirms the findings in the literature (Simeonidou and Vagiona 2018), namely that consumers are willing to pay an extra price for environmental friendly products, as 71% are willing to pay 5% more for their shopping cart in case the products they choose are produced with low water use. Also, 66.26% would choose an environmentally aware company for purchases even if it has more expensive products and 58.28% take into account the environmental image of the company in the products that they buy. The results from the above statements show that sustainable water use in production could be an effective marketing driver even if the product price is increased.

### Cross-sectional analysis of responses and demographical characteristics

**Fig. 8:**
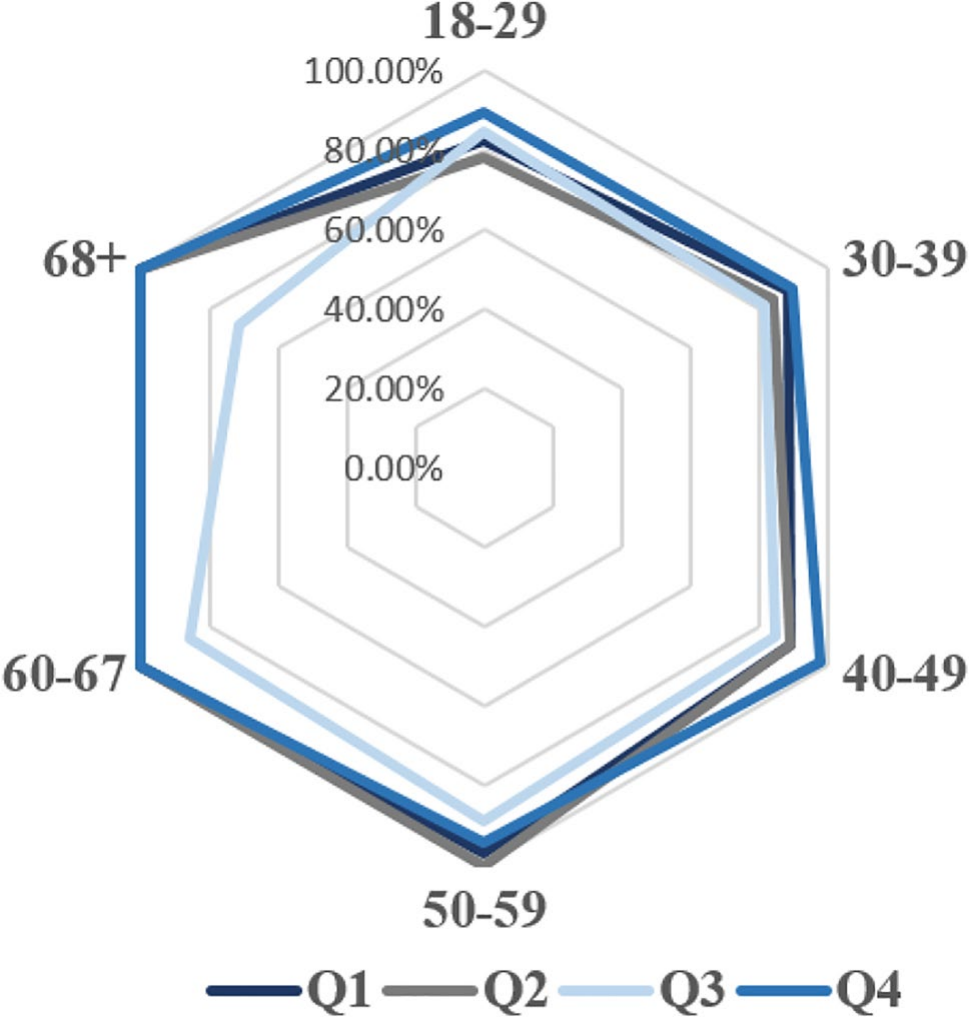
Percentage of the participants from each age group, who answered definitely yes or probably yes to the statements Q1, Q2, Q3 and Q4

In Fig. 8, the detailed results of the participants from each age group, who answered definitely yes or probably yes to the statements Q1, Q2, Q3 and Q4, are presented in a radar chart, widely used to display multivariate data. The results showed that people aged above 40 years are more aware of water use (Q1), they try to save water in their everyday life (Q2) and they recognize the importance of saving water in all sectors of the economy (e.g. industry) (Q4), as the 88% of the participants from these ages answered definitely yes or probably yes to these statements. Consumers aged 50–59 and 60–67 recognize that water will gradually become a non-renewable natural resource (Q3) in 88.89% and 68 + in 71.43%.

The results of this cross-sectional analysis indicate that consumers above 40 years old are more aware of environmental challenges and that education should play a leading role on increasing environmental awareness of the future generations.

**Fig. 9:**
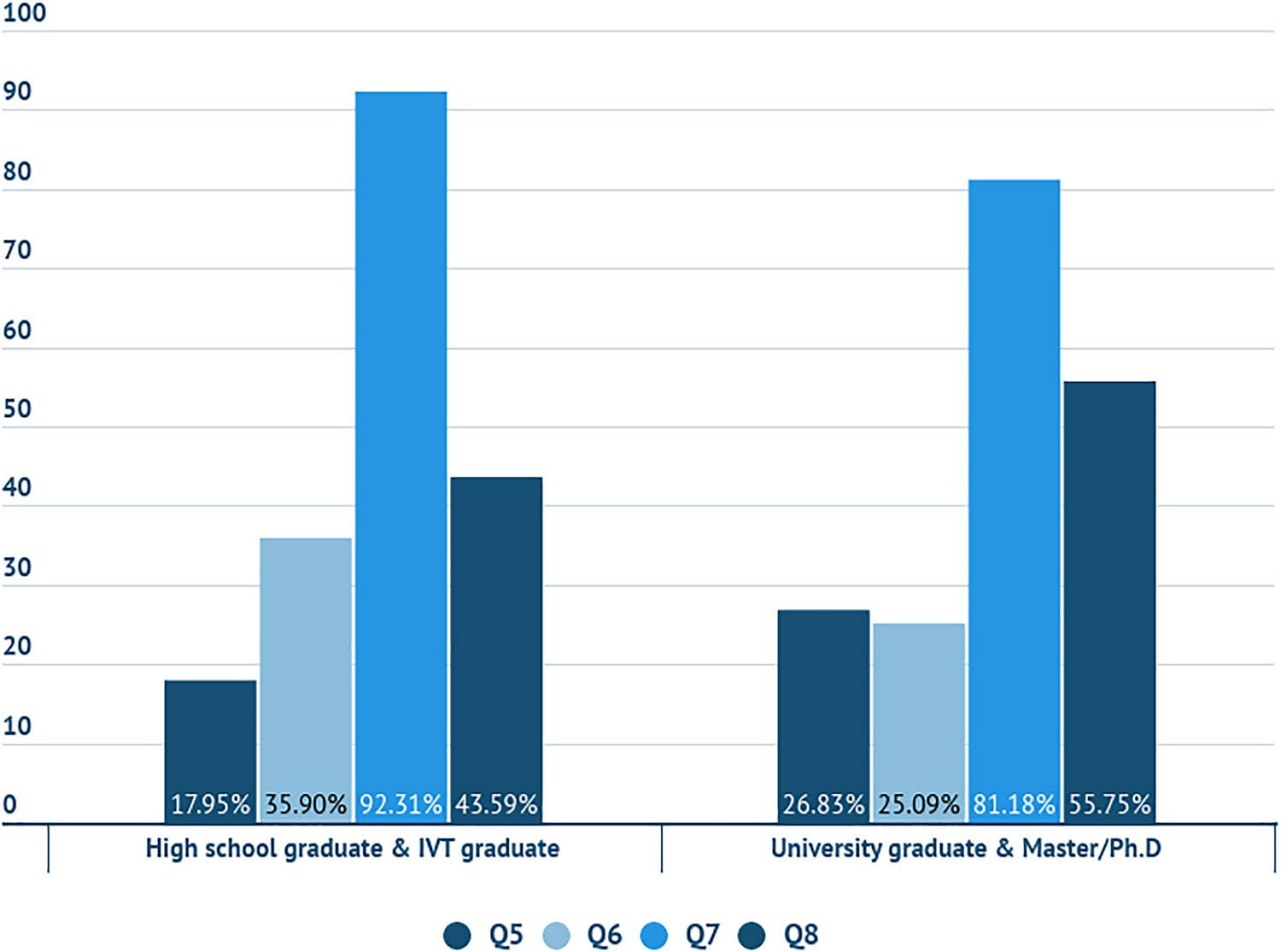
Percentage of the participants with different educational level, who answered definitely yes or probably yes to the statements Q5, Q6, Q7 and Q8

In Fig. 9, the percentage of the participants with different educational level, who answered definitely yes or probably yes to the statements Q5, Q6, Q7 and Q8, is presented. The results showed that few participants are aware that the water policy of the company is labelled in the product and would search additional information about it. Also half of participants with higher education (University graduates and Master/PhD) do not know what WF is, but at 81.18%, they would prefer a WF label on the product rather than the water policy of the company. Participants with secondary education (high school graduates and IVT graduates) would prefer a quantified label on the product at 92.31%, although only 43.59% knows what WF is (Fig. 8). It was noted that the educational level slightly affects the knowledge and preferences on water policies and labelling.

**Fig. 10:**
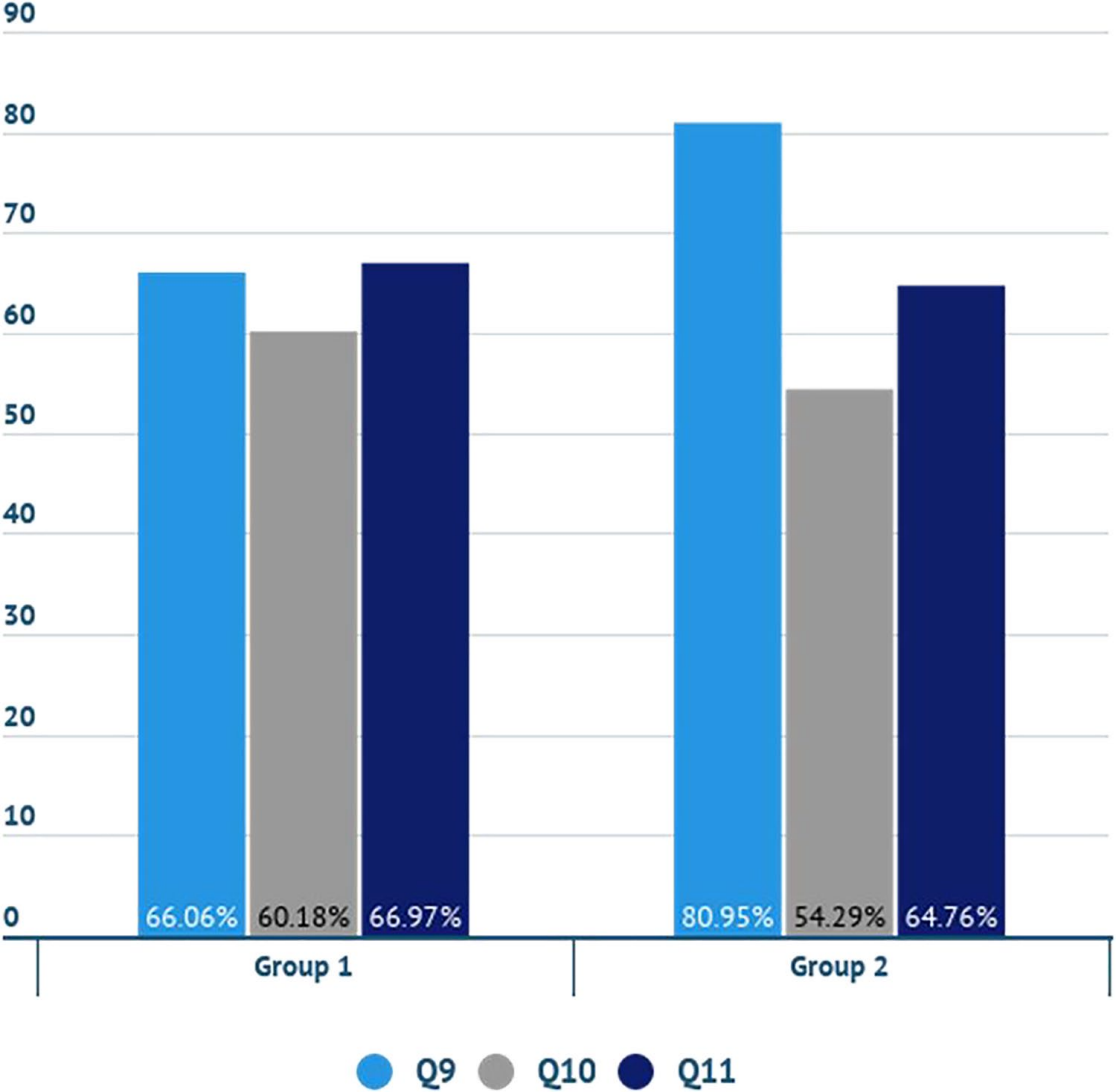
Percentage of the participants from group 1 and group 2, who answered definitely yes or probably yes to the statements Q9, Q10 and Q11

In Fig. 10, the percentage of the participants from group 1 and group 2, who answered “Definitely yes” or “Probably yes” to the statements Q9, Q10 and Q11, is presented. The work status of the participants and their willingness to pay for water sustainable products was expected to have a logical interrelation, as people with a stable job, i.e. PE, entrepreneurs and full time job (group 1), could be more willing to pay an extra price. However, the results of the survey showed that people with an unstable job, i.e. students, unemployed, farmers, part time job and retired (group 2), are more willing to pay 5% more for their shopping cart so that the products they choose are produced with low water use (Q9) compared to group 1. Consumers of group 1 take into account the environmental image of the company (Q10) in the product that they buy slightly more than consumers of group 2. The findings for Q11 are approximately the same for both groups.

The results of this cross-sectional analysis imply that the work status does not affect the willingness to pay more for sustainable products. Consumers with unstable job status are willing to pay more than people with a stable work status showcasing that environmental awareness and sustainable consumption are the main drivers for purchasing sustainable products.

## Conclusions

Following the results of the survey, from a diverse group of 326 people, labelling the water policy of the company cannot be considered an effective marketing driver for sustainability. Consumers are not aware that the water policy is labelled on the product and, if they knew, they would not search any additional information about it, despite of their awareness on water-related issues. Therefore, such type of information cannot communicate effectively the environmental awareness of the company concerning sustainable water consumption in production activities.

Most of the participants of the survey declared that they would prefer a quantified label of water consumption in the product. Through a WF label consumers could be informed instantly and effectively about water use for products’ manufacturing which will enable them to compare similar products and possibly choose products with lower water usage. The WF label could be also beneficial for companies as they can advertise their sustainable manufacturing practises in a more effective and client-oriented way. Nevertheless, consumers need to become more familiar with the WF concept, which could be achieved through publicity and marketing campaigns.

The cross-sectional analysis showed that young consumers (18–39 years old) are not quite aware of environmental challenges related to water, hence education should play a leading role on increasing environmental awareness of the future generations. It is also indicated that the preferences of a quantified water label and the level of knowledge on WF are not interconnected to the educational level of the participants.

Also, the majority of the participants are willing to pay an extra price for sustainable products and the cross-sectional analysis showed that job stability does not affect their purchases. Therefore, the company could benefit financially, as a large group of people is directed to a sustainable production and distribution corporate transition.

Comparing the results of the survey for the pro environmental behaviour of consumers towards WF labelling with the studies of consumers’ preferences concerning CF labels, many similarities and common tendencies can be observed. Firstly, age plays a critical role in both labels as consumers aged above 40 are more aware of environmental pressures and the need to take action. On the contrary, the educational level of participants in both WF and CF surveys does not affect their knowledge and understanding of WF labels which is very limited and does not influence their purchases. The job stability of consumers (criterion in our research) can ensure a high income (criterion in CF studies), so these two criteria could be compared in line with the willingness of consumers to pay for sustainable labelled products: in consumers’ preferences studies for CF labelled products it was noted that people with high income are more willing to pay a higher price, while on the current WF study job stability does not affect the purchase, as the majority of consumers (irrelevant of their job status) are willing to pay an extra price. This difference might be due to the importance of water as a basic resource to sustain life, leading consumers to easily accept higher prices.

Considering the available literature on CF labels and our findings on WF labelling, the readiness and willingness of consumers to accept and base their purchasing decisions on sustainability labels are strongly showcased. The adoption of a unified framework for CF labels and the introduction of a WF label for each industrial branch could thus support the wide adoption of these labels in products. Coupled with relevant educational programs and publicity campaigns to consumers to be more aware of the indicators and the type of information given, this introduction of these two labels could lead to higher public acceptance.

Finally, further research should be conducted to address a larger group of consumers with different characteristics (e.g. income), including the use of additional criteria on specific products, such as their necessity to the household, e.g. comparison of preferences between milk and chocolates and also analyse the impact of other labels for sustainability already applied in products, e.g. green dot, fair trade in consumers’ behaviour.

## Data Availability

The datasets generated and/or analysed during the current study are available from the corresponding author on reasonable request. The authors have no relevant financial or non-financial interests to disclose.
